# Plasma Catestatin Levels and Advanced Glycation End Products in Patients on Hemodialysis

**DOI:** 10.3390/biom11030456

**Published:** 2021-03-18

**Authors:** Mirko Luketin, Maja Mizdrak, Dijana Boric-Skaro, Dinko Martinovic, Daria Tokic, Marino Vilovic, Daniela Supe-Domic, Tina Ticinovic Kurir, Josko Bozic

**Affiliations:** 1 Department of Nephrology and Dialysis, University Hospital of Split, 21000 Split, Croatia; luketin.mirko@gmail.com (M.L.); mmizdrak@mefst.hr (M.M.); zanaboric@gmail.com (D.B.-S.); 2 Department of Pathophysiology, University of Split School of Medicine, 21000 Split, Croatia; d.m.993@hotmail.com (D.M.); marino.vilovic@mefst.hr (M.V.); tticinov@mefst.hr (T.T.K.); 3 Department of Anesthesiology and Intensive care, University Hospital of Split, 21000 Split, Croatia; dariatokic@gmail.com; 4 Department of Health Studies, University of Split, Split, Croatia, 21000 Split, Croatia; daniela.supedomic@gmail.com

**Keywords:** catestatin, advanced glycation end products, hemodialysis, Malnutrition-Inflammation Score, Dialysis Malnutrition Score

## Abstract

Catestatin (CST) is a pleiotropic peptide involved in cardiovascular protection with its antihypertensive and angiogenic effects. Considering that patients with end-stage renal disease (ESRD) who are undergoing hemodialysis (HD) are associated with higher cardiovascular risk, the aim of this study was to investigate plasma CST levels in HD patients, compare them to healthy controls and evaluate possible CST associations with advanced glycation end products (AGEs) and laboratory, anthropometric and clinical parameters. The study included 91 patients on HD and 70 healthy controls. Plasma CST levels were determined by an enzyme-linked immunosorbent assay in a commercially available diagnostic kit, while AGEs were determined using skin autofluorescence. Plasma CST levels were significantly higher in the HD group compared to the controls (32.85 ± 20.18 vs. 5.39 ± 1.24 ng/mL, *p* < 0.001) and there was a significant positive correlation between CST and AGEs (r = 0.492, *p* < 0.001). Furthermore, there was a significant positive correlation between plasma CST levels with both the Dialysis Malnutrition Score (r = 0.295, *p* = 0.004) and Malnutrition-Inflammation Score (r = 0.290, *p* = 0.005). These results suggest that CST could be playing a role in the complex pathophysiology of ESRD/HD and that it could affect the higher cardiovascular risk of patients on HD.

## 1. Introduction

End-stage renal disease (ESRD) is defined as an irreversible failure of kidney function. It represents stage 5 of chronic kidney disease (CKD) with an estimated glomerular filtration rate (eGFR) less than 15 ml per minute/1.73 m^2^ [1,2]. In recent decades, ESRD has grown to a global health concern, with the latest reports showing that the total number of ESRD patients is increasing rapidly [3,4]. This recent increase in prevalence is largely due to two factors: age and diabetes [5]. 

Hemodialysis (HD) is an artificial process which removes excess water and toxins from the blood. It is the most frequently used renal replacement therapy for ESRD patients [6]. With the recent rise in chronic kidney diseases and ESRD among them, HD is more frequently indicated. Even though it is indispensable for ESRD patients, it is associated with a high cardiovascular risk, peripheral neuropathy, parathyroid adenoma and acquired cystic disease of the kidney [7].

Advanced glycation end products (AGEs) represent an endogenously produced or exogenously derived group of substances which are formed through non-enzymatic glycation of lipids, proteins and nucleic acids with the Maillard reaction [8,9]. Since glycation is a constant process during aging and the rate at which glycated substances disintegrate is very slow, AGEs accumulate with rising age [10]. This process is aggravated by hyperglycemia, hyperlipidemia, oxidative stress and inflammation [11]. It has been proved that higher levels of AGEs are associated with diabetes [12], cardiovascular events [13], high BMI [14], CKD [15] and non-alcoholic fatty liver disease [16]. Endogenous and exogenous AGEs are partially degraded in the body and eliminated by kidneys which makes renal function both a target and an instigator for AGEs [17]. Since renal failure decreases the clearance of AGEs, it also initiates a vicious circle because increased AGE levels additionally impair renal function [18,19]. It was established that AGEs are overproduced in both peritoneal dialysis (PD) and HD settings. During the heat sterilization process of the PD fluids, glucose degradation products are accumulated in the peritoneum and they trigger the formation of AGEs in excessive amounts, leading to an increased oxidative stress [20]. During HD, it was shown that the reactive carbonyl compounds accumulated due to renal failure and the interaction of blood with the dialysis membrane, both contributing to an amplified formation of AGEs [15,21]. Consequential formation of oxidative products, loss of important antioxidants and chronic inflammation are the most significant factors that lead to high cardiovascular risk and increased mortality in CKD/HD settings [11,22]. 

The special interest of this study is catestatin (CST), a pleiotropic peptide which is formed endogenously by a proteolytic cleavage of the neuroendocrine hormone chromogranin A [23]. This novel peptide has shown different effects on homeostasis and various organs by suppression of catecholamine release and enhancement of histamine discharge from mast cells [24]. Recent studies reported its involvement in cardiovascular protection with its antihypertensive and angiogenic effects which could be used as a predictor for many disorders such as coronary artery disease, acute myocardial infarction, arrhythmia and heart failure [25,26,27,28,29,30,31,32]. Moreover, CST has been investigated in several studies with chronic inflammatory diseases such as inflammatory bowel disease and obstructive sleep apnea, where it showed its association with inflammation and possible immunomodulatory effects [33,34,35,36]. In a recent study, Sun et al. investigated a possible association between plasma CST levels and cardiovascular risk in hemodialyzed patients, and their results showed that CST might be an independent cardiac prognostic factor [37].

Besides the aforementioned study of Sun et al., the role of CST in CKD and HD settings is not well elaborated, so the aim of this study was to investigate plasma CST levels in patients undergoing HD and healthy controls. Additionally, we aimed to evaluate its possible association with AGEs and biochemical, anthropometric and clinical parameters.

## 2. Materials and Methods

### 2.1. Study Design and Ethical Considerations

This cross-sectional study was performed at the University Hospital of Split during the period from June 2018 to March 2019.

All subjects were informed about the procedures, purpose and course of the study, and every participant signed a written consent. The study was conducted in accordance with all ethical principles of the Seventh Revision of the Helsinki Declaration from 2013 and was approved by the Ethics Committee of University Hospital of Split.

### 2.2. Subjects

The study included 91 adult patients who were undergoing HD at the Department of Nephrology and Dialysis and 70 healthy controls. Inclusion criteria for HD participants were as follows: patients older than 18 years; undergoing HD for more than one year on a three-times-a-week program; stability during HD sessions; received dialysis dose (Kt/V) ≥ 1.2; body mass index (BMI) between 18.5 and 35 kg/m^2^; patients with a stable body weight and HbA1c < 9%. In the period of 3 months prior to the study, no subject in either group had a history of hospitalization, clinically evident cerebrovascular disease, unstable cardiovascular disease (acute coronary syndrome, heart failure), uncontrolled hypertension, severe anemia (hemoglobin level < 100 g/L), alcoholism, autoimmune diseases, malignancies and liver diseases. Furthermore, in the same 3-month period, none of the participants were receiving antibiotics, cytotoxic drugs, blood transfusions, corticosteroids or other medications which could interfere with the results. The aforementioned criteria were determined by reviewing participants’ medical records and interviewing them. All subjects who met the inclusion criteria underwent a screening test including a physical examination and review of clinical history, smoking status and alcohol consumption. 

The controls were recruited through the University Hospital of Split as volunteers and they were not compensated. After a detailed physical examination, screening and medical history review, we excluded all subjects who had a confirmed or suspected kidney disease. Kidney disease was considered absent if the eGFR (CKD-EPI (Chronic Kidney Disease Epidemiology Collaboration) creatinine equation) was > 60 mL/min/1.73 m^2^. 

All patients were undergoing intermittent HD, with bicarbonate dialysate at a flow rate of 375–450 mL/min and low-molecular weight heparin (LMWH) as standard anticoagulation using low-flux (ultrafiltration rate < 20 mL/mmHg/h) polysulfone membrane dialyzers FX8 and FX60 (Fresenius, Bad Homburg, Germany) with a blood flow rate of 250–300 mL/min. Temperature of dialysate was maintained at 36–37 °C and the dialysis bath consisted of bicarbonate 32–35 mmol/L, sodium 138–145 mmol/L, potassium 2 mmol/L and calcium 1.25–1.5 mmol/L. Ultrafiltration was measured volumetrically on the dialysis machine.

### 2.3. Blood Sampling and Laboratory Analysis

Blood samples were taken under fasting conditions just before connecting subjects to the dialysis machine and before giving them LMWH. The samples were taken according to standard laboratory practice where all the hematological and biochemical parameters were analyzed on the same day while samples for CST determination were stored for later analysis. Plasma CST levels were determined by an enzyme-linked immunosorbent assay (ELISA), by using a commercially available diagnostic kit (EK-053–27CE, EIA kit, Phoenix Pharmaceuticals Inc., Burlingame, CA, USA). According to the manufacturer’s instructions, the kit measurement range was 0–100 ng/mL. Reported sensitivity for CST was 0.05 ng/mL with a linear range of 0.05–0.92 ng/mL. Cross-reactivity with endogenous human CST peptide for this assay kit was 100% with the intra-assay and inter-assay coefficients of variability being < 10% and < 15%, respectively. All blood samples were managed according to international standards, in the same laboratory, by the same experienced medical biochemist. Moreover, the biochemist was blinded to the subjects’ group in the study. Other biochemical parameters were analyzed according to standard laboratory procedures. Creatinine, urea and potassium levels were analyzed once more after the dialysis in the HD group.

### 2.4. Blood Pressure Measurement

Blood pressure of the participants was measured prior the HD and right after the HD by an experienced nephrologist. The Riester aneroid sphygmomanometer (Rudolf Riester GmbH, Jungingen, Germany) and 3M Littmann Classic III stethoscope (3M, Saint Paul, US) were used for measurements. The participants were first seated for 15 minutes after which the inflatable sphygmomanometer’s cuff was placed on the upper arm just above the elbow pit for three consecutive measurements. Mean of three measurements was calculated to achieve an accurate blood pressure value. 

### 2.5. AGEs Measurement

AGEs were determined using skin autofluorescence (SAF), which was measured using the AGE-Reader SU (DiagnOptics Technologies BV, Groningen, The Netherlands). SAF was calculated as the average light intensity in the 420–600 nm range divided by the average light intensity in the 300–420 nm range, and the result was expressed in arbitrary units (AU). Measurements of SAF were performed on the 4 cm^2^ surface of the lower arm prior to the anthropometric measurements. The dominant forearm of patients was positioned on the device for three consecutive measurements and patients were instructed not to use skin creams or lotions before the assessment. Mean of three measurements was calculated to achieve an accurate SAF value. Furthermore, the AGE-Reader determined the cardiovascular risk of the subjects according to their AGEs values in regard to the subjects’ age.

### 2.6. Anthropometric Measurements and Clinical Examination

All participants underwent full physical examination and measurements of anthropometric characteristics: body weight, body height, BMI, waist circumference and hip circumference. A calibrated medical scale with built-in heights (Seca, Birmingham, UK) was used for measurement of body weight and height. BMI was calculated according to the formula = [body weight (kg)]/[height per square (m^2^)]. The middle distance between the bottom of the rib cage in the middle axillary line and the tip of the iliac crest in the standing upright position of the examinee was used for measurement of waist circumference. The circumference of the hips was measured at the level of the largest circumference of the gluteal muscles, above the line connecting the large trochanters of the femur.

### 2.7. Malnutrition and Inflammation Assessment Scores

Two validated grading systems were used to assess the nutritional and inflammation statuses of the patients undergoing HD. The evaluations were conducted by the same trained physician within 5 to 15 minutes before anthropometric measurements. 

The Malnutrition-Inflammation Score is a validated and reliable quantitative score for assessing the presence and evaluating the degree of nutritional deficit in dialysis patients. It has 10 elements (change in end dialysis; dietary intake; gastrointestinal symptoms; functional capacity; comorbidity and years on dialysis; decreased fat stores; signs of muscle wasting; BMI; serum albumin; serum TIBC), each with four levels of severity, from 0, normal, to 3, severely abnormal. The sum of all ten MIS levels ranges from 0 to 30, with a higher score indicating a more severe malnutrition and inflammation [38]. 

The Dialysis Malnutrition Score is a score used for evaluation of nutritional status in dialysis patients. It consists of 7 components (weight change; dietary intake; gastrointestinal symptoms; functional capacity; comorbidity; decreased fat stores; signs of muscle wasting) and each one has a score between 1, normal, and 5, severely abnormal. The sum of all seven DMS components ranges from 7 to 35, with a higher score indicating a greater degree of malnutrition [39].

### 2.8. Statistical Analysis

All data were analyzed with statistical software MedCalc (MedCalc Software, Ostend, Belgium, version 17.4.1). Quantitative data were expressed as mean ± standard deviation or median and interquartile range, while qualitative data were expressed as whole number and percentage. The normality of data distribution was estimated with the Kolmogorov–Smirnov test. Student’s *t*-test for independent samples and the Mann–Whitney U test were used for comparison of plasma CST levels and other parameters between patients undergoing HD and the control subjects. The chi-square test was used for comparison of qualitative variables. The correlation between biochemical, anthropometric and clinical parameters with plasma CST levels was estimated with Pearson’s correlation or Spearman’s correlation. Furthermore, the HD group was divided into tertiles depending on their AGE level, and corresponding plasma CST levels were calculated and compared using one-way ANOVA with the post hoc Sceffé test. Lastly, multiple linear regression analysis adjusted for age, gender, anthropometric measurements, HD duration, MIS, DMS and AGEs was used to determine significant independent predictors of plasma CST levels. For this analysis, we reported the corresponding *p*-values with unstandardized β-coefficients, standard error and t-values. The level of statistical significance in this study was set at *p* < 0.05.

## 3. Results

### 3.1. Baseline Characteristics of the Study Population

There were no statistically significant differences between the HD group and the control group regarding age, gender, anthropometric measures and systolic and diastolic blood pressures. In the HD group, the median CKD duration was 11 (7.0–26.5) years and the median HD duration was 4 (2.0–8.0) years. Furthermore, the mean Dialysis Malnutrition Score (DMS) was 13.8 ± 4.04 and the mean Malnutrition-Inflammation Score (MIS) was 7.17 ± 3.88 (Table 1).

### 3.2. Laboratory Parameters of the Study Population

There were statistically significant differences between the HD group and the control group in the laboratory parameters, as the patients undergoing HD had a higher level of C-reactive protein (CRP) (*p* < 0.001), fasting glucose (*p* = 0.003), phosphates (*p* < 0.001), PTH (*p* < 0.001), uric acid (*p* = 0.005), creatinine (*p* < 0.001) and urea (*p* < 0.001). Moreover, the control group had a significantly higher level of erythrocytes (*p* < 0.001), hemoglobin (*p* < 0.001), hematocrit (*p* < 0.001), plasma iron (*p* < 0.001), potassium (*p* < 0.001), total proteins (*p* < 0.001), albumins (*p* < 0.001) and sodium (*p* = 0.024) (Table 2).

### 3.3. Skin Autofloruscence Level of AGEs in the Study Population

**Figure 1:**
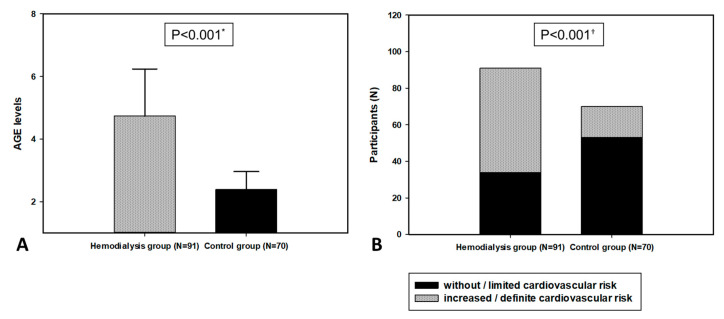
Advanced glycation end product (AGE) levels in hemodialysis and control groups (**A**) and categories of cardiovascular risk according to AGE levels in study population (**B**). Abbreviations: AGEs, advanced glycation end products. *, *t*-test for independent samples. †, chi-square test.

The level of AGEs was significantly higher in the HD group compared to the control group (4.74 ± 1.50 vs. 2.38 ± 0.57, *p* < 0.001). Furthermore, according to AGE levels in the study population, there were significantly more patients with an increased cardiovascular risk in the HD group compared to the control group (57 (77%) vs. 34 (39.1%), *p* < 0.001) (Figure 1).

### 3.4. Plasma CST Levels in the Study Population

**Figure 2:**
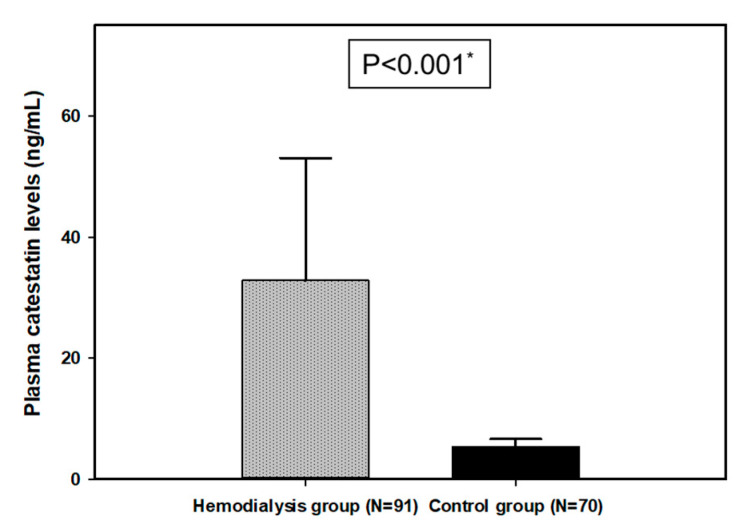
Plasma catestatin (CST) levels in hemodialysis (N = 91) and control group (N = 70) participants. *, *t*-test for independent variables.

Plasma CST levels were significantly higher in the HD group compared to the control group (32.85 ± 20.18 ng/mL vs. 5.39 ± 1.24 ng/mL, *p* < 0.001) (Figure 2).

### 3.5. CST and AGEs Correlations with Anthropometric, Laboratory and Clinical Parameters in the HD Group

There was a significant positive correlation between CST and age (r = 0.290, *p* = 0.005) and there were significant negative correlations between CST and both pre-dialysis creatinine (r = −0.245, *p* = 0.019) and post-dialysis creatinine (r = −0.225, *p* = 0.032). Moreover, there was a significant negative correlation between AGEs and pre-dialysis urea (r = −0.216, *p* = 0.039), post-dialysis urea (r = −0.241, *p* = 0.021), LDL-cholesterol (r = −0.214, *p* = 0.041), triglycerides (r = −0.211, *p* = 0.045) and BMI (r = −0.249, *p* = 0.017) (Table 3). There was no statistically significant correlation with the other parameters.

### 3.6. Correlation between CST and AGEs

**Figure 3:**
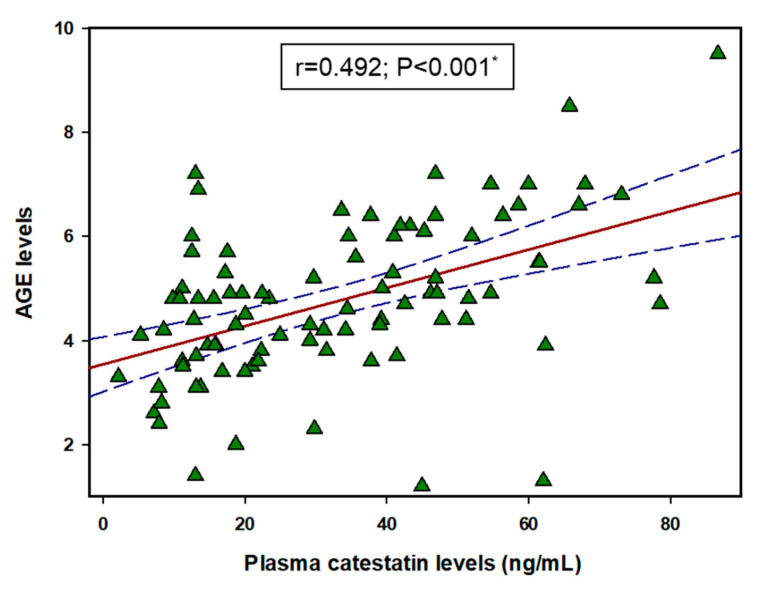
Correlation between CST and AGE levels in hemodialysis patients (N = 91). Abbreviations: AGE, advanced glycation end product. *, solid red line represents Pearson’s correlation coefficient, while blue lines represent 95% confidence intervals.

**Figure 4:**
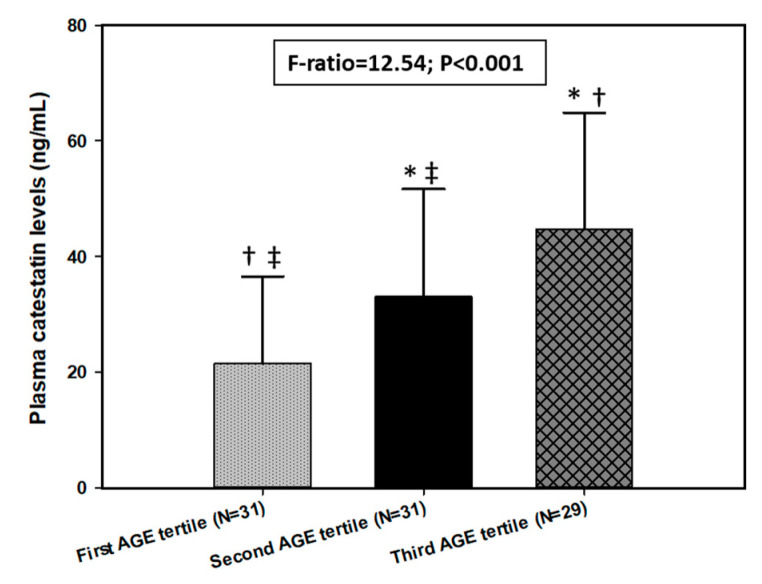
CST levels between tertiles of AGE levels. Abbreviations: AGE, advanced glycation end product. Tested with one-way analysis of variance (ANOVA) with post hoc Sceffé test to examine differences between each of the groups. *, *p* < 0.05 vs. first AGE tertile. †, *p* < 0.05 vs. second AGE tertile. ‡, *p* < 0.05 vs. third AGE tertile.

There was a significant positive correlation between CST and AGEs (r = 0.492, *p* < 0.001) (Figure 3). Furthermore, after dividing the HD group into tertiles depending on their AGE levels, there was a statistically significant difference between each tertile in plasma CST level (first: 21.50 ± 15.05 ng/mL vs. second: 33.04 ± 18.64 ng/mL vs. third: 44.79 ± 20.08 ng/mL, F = 12.54; *p* < 0.001) (Figure 4).

### 3.7. CST and AGEs Correlation with MIS and DMS

**Figure 5:**
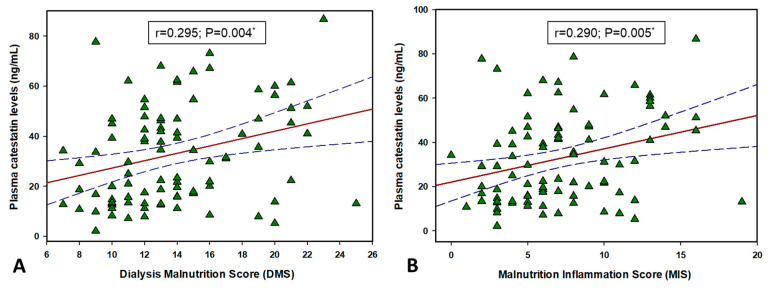
Correlation between CST levels and Dialysis Malnutrition Score (**A**) and Malnutrition-Inflammation Score (**B**) in hemolysis patients (N = 91). *, solid red line represents Pearson’s correlation coefficient, while blue lines represent 95% confidence intervals.

**Figure 6:**
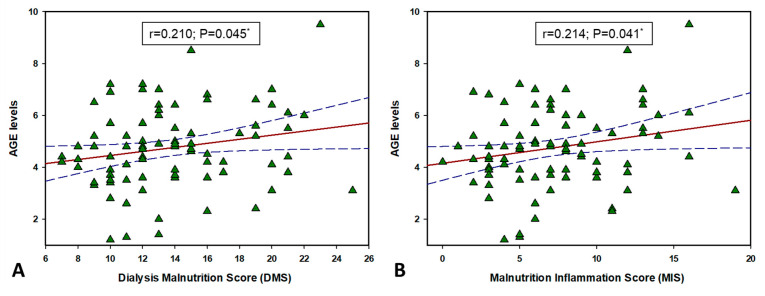
Correlation between AGE levels and Dialysis Malnutrition Score (**A**) and Malnutrition-Inflammation Score (**B**) in HD patients (N = 91). Abbreviations: AGE, advanced glycation end product. *, solid red line represents Pearson’s correlation coefficient, while blue lines represent 95% confidence intervals.

There was a significant positive correlation between plasma CST levels with both DMS (r = 0.295, *p* = 0.004) and MIS (r = 0.290, *p* = 0.005) (Figure 5). Additionally, there was also a significant positive correlation between AGE levels and both DMS (r = 0.210, *p* = 0.045) and MIS (r = 0.214, *p* = 0.041) (Figure 6).

### 3.8. Multiple Linear Regression Model for Plasma CST Levels

Multiple linear regression analysis showed that plasma CST levels as a dependent variable retained a significant association with age (β ± SE, 0.31 ± 0.14, *p* = 0.042), waist circumference (0.49 ± 0.24, *p* = 0.048) and AGE levels (5.20 ± 1.25, *p* < 0.001) after model adjustment for gender, age, BMI, waist circumference, MIS, DMS, AGE levels and HD duration (Table 4).

## 4. Discussion

This study showed that the levels of both CST and AGEs are significantly higher in patients undergoing HD compared to the healthy control group. That is in line with the outcomes of a recent cohort study conducted by Sun et al., which also showed significantly higher levels of CST in patients undergoing HD compared to the healthy controls [32]. Moreover, their study showed that CST could be an independent predictor of cardiovascular mortality as they found that patients with higher plasma CST levels had more cardiac deaths in comparison to their counterparts. These results support the well-established association of long-term HD with cardiovascular complications, such as arrythmia, hypertension and heart failure [40,41]. 

HD is associated with a low-grade chronic inflammation and oxidative stress which is due to loss of antioxidants and stimulation of leukocytes during dialysis which triggers production of reactive oxygen species (ROS) [42,43,44]. Several recent studies suggested that that protective role of CST is mediated by preventing DNA damage caused by ROS, reducing endoplasmic reticulum stress-induced apoptosis and scavenging free radicals [45,46,47,48], while the study conducted by Aung et al. implied that CST also has an immunomodulatory function, as they determined that CST has a role in inflammation by causing mast cells to release leukotrienes, prostaglandins, cytokines and chemokines [49]. Another recent cohort study on patients with heart failure with reduced ejection fraction (HFrEF) showed that CST levels could predict all-cause and unplanned hospitalizations [50]. All of this suggests that CST could have a role in the complex pathophysiology of patients undergoing HD. 

However, it is still unclear whether the observed CST increase is part of the protective feedback loop mechanism in the state of chronic inflammation, oxidative stress and hypertension, or whether CST is one of the causative factors in the pathophysiology of these disorders. CST is a well-established inhibitor of the catecholamine secretion with an effect on the autonomic nervous system which could explain its possible influence on the blood pressure and its link with cardiovascular incidents [24,51,52,53]. Still, it is important to note that several studies showed a strong association of renal failure with high levels of CST’s precursor chromogranin A. Moreover, a recent study even determined an association of a chromogranin A gene polymorphism with hypertensive renal disease [54,55,56,57], while the study by Chen Y. et al. showed that chromogranin A, although considered a regulator of catecholamine storage and release, also appears to have a role in endothelial function and cell actions in the glomeruli [58]. Nonetheless, more studies are needed to address the complex relationship between CST and chromogranin A in CKD/HD settings. 

Another major finding of this study is the significant positive correlation between AGEs and CST. AGEs are a group of different products made by non-enzymatic glycation of sugars, lipids and proteins, forming and decomposing in kidneys [59]. They accumulate with rising age and contributing factors such as hyperglycemia, hyperlipidemia, oxidative stress and other illnesses which affect the renal system [60,61]. Several studies pointed to a strong association between oxidative stress and the level of AGEs [20,21,22]. Moreover, with AGEs’ association with inflammation, oxidative stress and uremic state due to CKD, it could be hypothesized that all of these factors cumulatively contribute to cardiovascular mortality and morbidity in CKD/HD patients. The results of this study show that the levels of AGEs are significantly higher in HD patients compared to the control group, which is in alignment with the well-established relationship between AGEs and CKD. It is recognized that the accumulation of AGEs accelerates atherosclerosis through cross-linking of the key proteins for endothelial and platelet function while also glycating LDL which makes it more prone to oxidation and further enhancement of atherosclerosis, and all of this could gradually lead to a higher risk of cardiovascular incidents [62,63,64]. This is consistent with the result of several studies which emphasized the association between a high level of AGEs and cardiovascular morbidity and mortality in ESRD patients [65,66,67]. CKD is a known cause of cardiovascular complications and deaths due to its connection to sympathetic overactivity, blood vessel stiffness and structural myocardial changes [68]. On the other hand, although being a therapy for CKD, HD, as previously mentioned, also causes further damage by engaging in oxidative stress through insufficient removal of ROS, frequent disbalance in electrolytes and disproportionate levels of intravascular fluids during sessions of dialysis [69,70,71,72]. The results of this study also show that patients undergoing HD have a significantly higher cardiovascular risk determined by the level of AGEs, compared to the healthy controls. As it is established that CST is increased in states of elevated oxidative stress and low-grade chronic inflammations such as CKD, and that the AGEs accumulation process is elevated in those same conditions, these two factors combined could have a significant predictive role of cardiovascular complications and incidents in patients who are undergoing HD. After dividing the HD group into tertiles depending on their AGE levels, we found a significant difference in plasma CST levels between each of them, with the first AGEs tertile having the lowest and the third AGEs tertile having the highest values of plasma CST. Likewise, the multiple linear regression further supports this evidence as it showed that CST retained a significant association with AGEs after model adjustment for gender, age, BMI, HD duration, MIS and DMS. 

Other significant results of this study are the correlations of CST and AGEs with both MIS and DMS scores. Malnutrition–inflammation complex syndrome (MICS) is the protein–energy malnutrition and proinflammatory state which occurs in patients undergoing HD [73]. Since the presence of MICS in HD patients is associated with anorexia, atrophy, anemia and possibly accelerated atherosclerosis, this condition is a strong predictor of morbidity and mortality in HD patients [74,75]. MIS and DMS are the two most comprehensive scoring systems for evaluating malnutrition and inflammation in HD patients and as such they have a strong correlation with the clinical outcome [33,34]. While these results demonstrate a significant association of CST and AGEs with nutrition and inflammation scores in HD patients, the causal–consequential relations could not be established in this study. Specifically, CST and AGEs increase could contribute to the development of inflammation and malnutrition but could also accompany worse cardiovascular condition in the undernourished HD patients. However, it has been shown by several studies that CST can interfere with the major cerebral neuroreceptor-blocking sites such as the GABAergic system, especially in cerebral areas that are involved in feeding behaviors [26,76]. Furthermore, in a recent animal study conducted on hamsters, there was a strong anorexigenic response following treatment with CST as it induced a significant reduction in eating and drinking behaviors [77]. It is possible that the high level of CST in HD patients is affecting their eating behaviors through the inhibition of complex neuroreceptor-blocking sites, as proposed by these several studies, and that CST could be one of the links between malnutrition and CKD. 

The limitation of this study is its cross-sectional design which does not allow the determination of any causal relationship. Additionally, the sample size was relatively small and we were not able to completely eliminate all the possible confounding effects. A future larger-scale, multicenter longitudinal study needs to address these findings.

In conclusion, this study indicates a potential link between CST and CKD/HD as well as a positive correlation between CST and AGEs. Furthermore, these results imply that CST could be involved in the cardiovascular pathophysiology of HD patients and could possibly predict cardiovascular incidents in these patients. 

## Figures and Tables

**Table 1:** Baseline parameters of the study population.

Parameter	Hemodialysis Group (N = 91)	Control Group (N = 70)	*p* *
Male gender	58 (63.7)	34 (68.0)	0.612
Age (years)	68.3 ± 12.6	67.2 ± 12.1	0.577
Body weight (kg)	74.9 ± 14.9	75.3 ± 14.7	0.865
Body height (cm)	174.1 ± 9.6	176.3 ± 8.1	0.125
Body mass index (kg/m^2^)	24.5 ± 4.8	23.9 ± 3.8	0.391
Waist circumference (cm)	98.7 ± 12.5	96.1 ± 11.1	0.171
Hip circumference (cm)	103.3 ± 10.7	100.6 ± 15.6	0.195
Pre-dialysis systolic pressure (mmHg)	135.1 ± 27.4	−	−
Post-dialysis systolic pressure (mmHg) ^‡^	123.2 ± 27.5	118.5 ± 7.1	0.117
Pre-dialysis diastolic pressure (mmHg)	70.1 ± 16.1	−	−
Post-dialysis diastolic pressure (mmHg) ^‡^	69.3 ± 14.7	72.1 ± 8.2	0.137
Diabetes	29 (31.9)	14 (20.0)	0.091
Smoking	28 (30.8)	24 (34.3)	0.636
CKD duration (years) ^†^	11 (7.0–26.5)	−	−
Hemodialysis duration (years)	4.0 (2.0–8.0)	−	−
Urine output (mL)	200.0 (0–1075.0)	−	−
Erythropoietin therapy	77 (84.6)	−	−
Arteriovenous fistula #	48 (52.7)	−	−
Temporary CVC #	27 (29.7)	−	−
Permanent CVC #	16 (17.6)	−	−
Dialysis Malnutrition Score	13.8 ± 4.0	−	−
Malnutrition-Inflammation Score	7.1 ± 3.8	−	−

**Abbreviations: CKD**, chronic kidney disease; **CVC**, central venous catheter. Data are presented as mean ± standard deviation, median (IQR) or N (%). *, *t*-test for independent samples, Mann–Whitney U test or chi-square test. ^‡^, post-dialysis stands only for the hemodialysis (HD) group. ^†^, period of time from the initial CKD diagnosis. #, venous approaches.

**Table 2:** Laboratory findings of hemodialysis patients.

Parameter	Hemodialysis Group (N = 91)	Control Group (N = 70)	*p* *
Erythrocytes (×10^12^/L)	3.7 ± 0.5	4.3 ± 0.6	<0.001
Hemoglobin (g/L)	110.7 ± 10.6	137.5 ± 11.9	<0.001
Hematocrit	0.34 ± 0.03	0.42 ± 0.05	<0.001
MCV (fL)	91.9 ± 6.9	92.2 ± 6.2	0.776
Plasma iron (µmol/L)	11.4 ± 5.2	16.8 ± 4.8	<0.001
Total proteins (g/L)	65.9 ± 5.2	71.5 ± 5.7	<0.001
Albumins (g/L)	38.5 ± 3.0	43.1 ± 2.8	<0.001
Pre-dialysis creatinine (µmol/L)	833.8 ± 186.1	−	−
Post-dialysis creatinine (µmol/L)^‡^	335.5 ± 103.5	82.8 ± 16.8 ^†^	<0.001
Pre-dialysis urea (mmol/L)	23.8 ± 6.2	−	−
Post-dialysis urea (mmol/L) ^‡^	8.1 ± 3.5	6.2 ± 2.1 ^†^	<0.001
Pre-dialysis potassium (mmol/L)	5.2 ± 0.8	−	−
Post-dialysis potassium (mmol/L) ^‡^	3.5 ± 0.3	4.3 ± 0.4 ^†^	<0.001
Uric acid (µmol/L)	345.2 ± 74.6	312.5 ± 68.4	0.005
Sodium (mmol/L)	138.4 ± 3.2	139.6 ± 3.4	0.024
Calcium (mmol/L)	2.27 ± 0.17	2.32 ± 0.12	0.038
Phosphates (mmol/L)	1.6 ± 0.5	1.1 ± 0.3	<0.001
Chlorides (mmol/L)	101.9 ± 3.2	102.2 ± 3.3	0.566
PTH (pmol/L)	37.0 (17.0–56.0)	5.0 (4.0–6.0)	<0.001
Fasting glucose (mmol/L)	6.7 ± 3.1	5.5 ± 0.7	0.003
CRP (mg/L)	4.5 (1.5–8.5)	0.8 (0.4–1.8)	<0.001
Total cholesterol (mmol/L)	4.4 ± 3.1	4.6 ± 2.9	0.677
HDL-cholesterol (mmol/L)	1.08 ± 0.36	1.12 ± 0.24	0.424
LDL-cholesterol (mmol/L)	2.2 ± 1.0	2.4 ± 1.1	0.109
Triglycerides (mmol/L)	1.96 ± 1.04	1.84 ± 0.98	0.458

Abbreviations: MCV, mean corpuscular volume; PTH, parathyroid hormone; CRP, C-reactive protein; HDL, high-density lipoprotein; LDL, low-density lipoprotein. Data are presented as mean ± standard deviation or median (IQR). *, *t*-test for independent samples or Mann–Whitney test. ^†^, fasting values for control group. ^‡^, post-dialysis stands only for the HD group.

**Table 3:** Correlation of CST and AGE levels in hemodialysis patients between selected anthropometric, laboratory and clinical parameters (N = 91).

Parameter	CST r (*p* *)	AGE r (*p* *)
Total proteins (g/L)	−0.083 (0.432)	−0.210 (0.045)
Albumins (g/L)	−0.159 (0.133)	−0.167 (0.113)
Pre-dialysis creatinine (µmol/L)	−0.245 (0.019)	−0.048 (0.651)
Post-dialysis creatinine (µmol/L)	−0.225 (0.032)	−0.112 (0.288)
Pre-dialysis urea (mmol/L)	−0.198 (0.060)	−0.216 (0.039)
Post-dialysis urea (mmol/L)	−0.143 (0.176)	−0.241 (0.021)
Pre-dialysis potassium (mmol/L)	−0.094 (0.373)	−0.002 (0.983)
Post-dialysis potassium (mmol/L)	−0.122 (0.250)	−0.205 (0.051)
CRP (mg/L)	−0.086 (0.419)	0.033 (0.758)
Total cholesterol (mmol/L)	0.170 (0.108)	0.164 (0.121)
HDL-cholesterol (mmol/L)	0.002 (0.988)	0.098 (0.354)
LDL-cholesterol (mmol/L)	0.040 (0.706)	−0.214 (0.041)
Triglycerides (mmol/L)	−0.106 (0.319)	−0.211 (0.045)
Pre-dialysis systolic pressure (mmHg)	−0.059 (0.577)	−0.069 (0.514)
Post-dialysis systolic pressure (mmHg)	−0.009 (0.931)	0.112 (0.289)
Pre-dialysis diastolic pressure (mmHg)	0.018 (0.865)	0.021 (0.846)
Post-dialysis diastolic pressure (mmHg)	0.084 (0.427)	0.107 (0.311)
Age (years)	0.290 (0.005)	0.116 (0.272)
CKD duration (years)†	0.089 (0.399)	0.111 (0.296)
Hemodialysis duration (years)	0.068 (0.524)	0.060 (0.575)
Body mass index (kg/m^2^)	−0.172 (0.103)	−0.249 (0.017)
Waist circumference (cm)	0.066 (0.531)	−0.084 (0.426)

Abbreviations: AGE, advanced glycation end product; CRP, C-reactive protein; HDL, high-density lipoprotein; LDL, low-density lipoprotein; CKD, chronic kidney disease. *, Pearson’s correlation test or Spearman’s correlation test. †, period of time from the initial CKD diagnosis.

**Table 4:** Multiple linear regression model of independent predictors for CST concentration.

Variable	β *	SE ^†^	*t*-Value	*p*
Gender	−3.36	28.1	−0.85	0.394
Age (years)	0.31	0.14	2.06	0.042
Body mass index (kg/m^2^)	−0.97	0.65	0.14	0.139
Waist circumference (cm)	0.49	0.24	2.0	0.048
Hemodialysis duration (years)	0.25	0.24	1.04	0.299
Malnutrition-Inflammation Score	0.95	1.11	0.85	0.393
Dialysis Malnutrition Score	−0.01	1.05	−0.01	0.986
AGE levels	5.20	1.25	4.16	<0.001

Abbreviations: AGE, advanced glycation end product. *, unstandardized coefficient β. ^†^, standard error.

## Data Availability

All data are available from the corresponding author. You can contact him via email: josko.bozic@mefst.hr.
